# Supplementary material for: CT in relation to RT-PCR in diagnosing COVID-19 in The Netherlands: A prospective study
Source: PLoS One. 2020 Jul 9;15(7):e0235844. doi: 10.1371/journal.pone.0235844 (PMC7347219; doi:10.1371/journal.pone.0235844)
Supplement: Attachment — Submitted filename: Reviewer Attachments for Manuscript Number PONE-D-20-13081.docx [file pone.0235844.s002.docx]

Appraised by a revised tool for the quality assessment of diagnostic accuracy studies (QUADAS2), your research is qualified. Due to some limitation of PCR assay, the accuracy of chest CT as a diagnostic tool for COVID-19 is an important issue during the progress of pandemic. Although there were some published reports in this topic, we still need more studies to clarify the certainty of evidences especially from the countries other than China. Therefore, it is worth to publish your research before accomplishing the answers of the below issues.

1. Did you calculate the power for your diagnostic study by statistical software based upon the prospective design of this research? If no or insufficient power, please mention it as one of the limitations of your study.
2. You did not introduce the software for statistical analysis. You should demonstrate the 95% confidence interval of sensitivity, specificity, likelihood ratio, and other results.
3. You performed some sensitivity analyses including CURB-65 score and SOFA score to distinguish the different diagnostic value in different severity of patients. Is it possible to perform more sensitivity analyses toward CT findings? You mentioned that “Chest CT can show characteristic findings including areas of ground-glass, with or without signs of reticulation (so called “crazy paving pattern”), consolidative pulmonary opacities in advanced stages and the “reverse halo” sign. Since peripheral areas of ground glass are a hallmark of early COVID-19, which can easily be missed at chest X-rays, CT scanning has an advantage over chest X-rays in the early stages of COVID-19.” Can you calculate the diagnostic values of above characteristic findings in CT?
4. You described the detailed methods very well. You mentioned that “ The chest CT was obtained in a mobile CT scan unit (Alliance Medical equipped with GE148 lightspeed 16 slice scanner) upon arrival at the ED.” Please introduce more details about how to avoid nosocomial contamination in your policies.
