# Supplementary material for: CT in relation to RT-PCR in diagnosing COVID-19 in The Netherlands: A prospective study
Source: PLoS One. 2020 Jul 9;15(7):e0235844. doi: 10.1371/journal.pone.0235844 (PMC7347219; doi:10.1371/journal.pone.0235844)
Supplement: Attachment — Submitted filename: Plos one CT COVID-19_revision decision.docx [file pone.0235844.s004.docx]

The authors have adequately addressed the reviewers’ comments. The revised manuscript is qualified and acceptable for publication in the *PLOS ONE*.
